# Supplementary figures and images for: Brief report: Lymph node morphology in stage II colorectal cancer
Source: PLoS One. 2021 Mar 29;16(3):e0249197. doi: 10.1371/journal.pone.0249197 (PMC8007027; doi:10.1371/journal.pone.0249197)

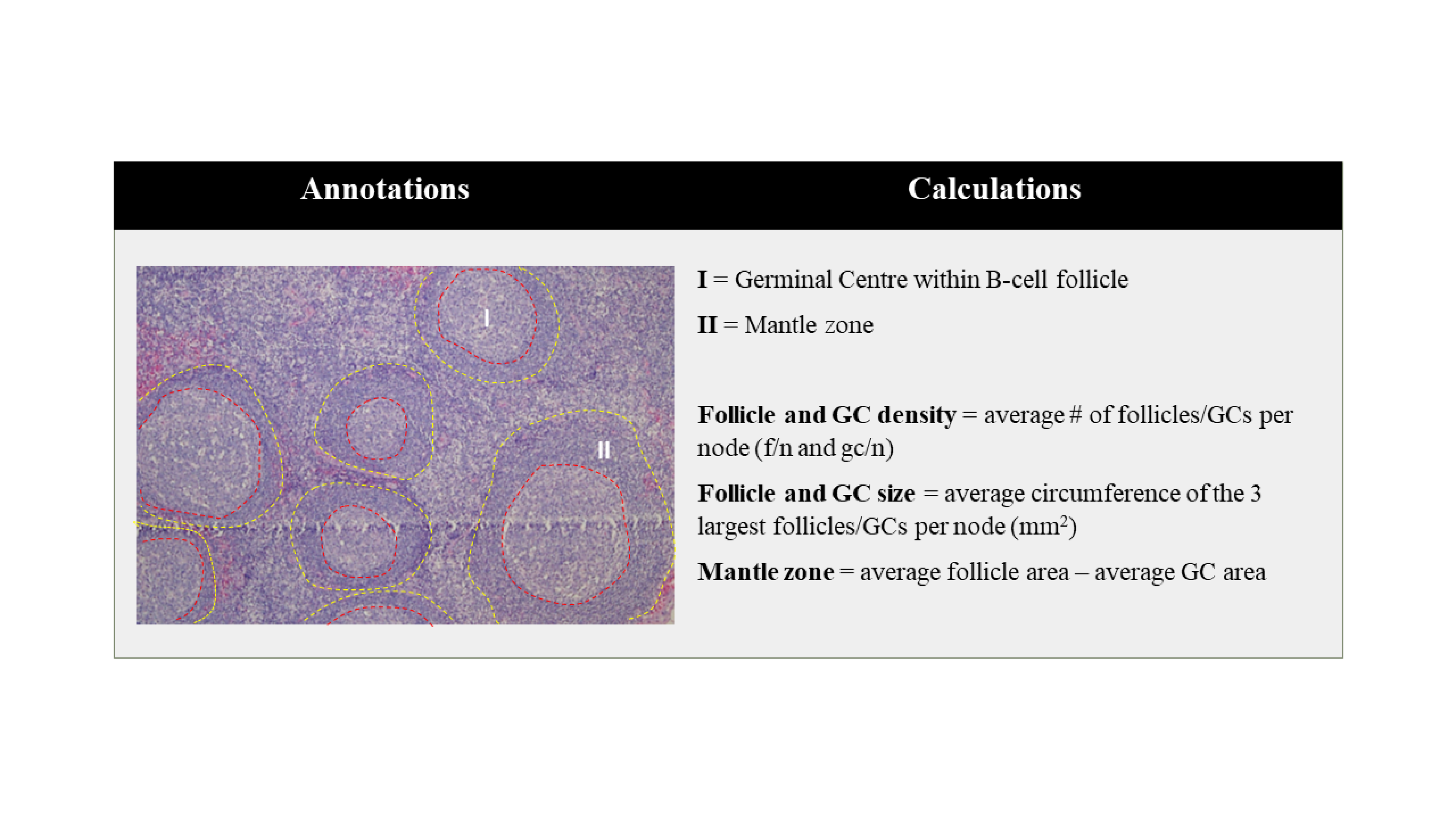

Supplement: S1 Fig — Evaluation is modified from a previously published method [7]. Left: All lymph nodes were annotated for B cell compartments (red and yellow dashed lines) using ImageJ. (I) Circular Germinal centre (GCs) within B-cell follicle, (II) Mantle zone. Right: Calculations for follicle and GC density, follicle and GC size, and mantle zone. (TIF) [file pone.0249197.s001.tif]

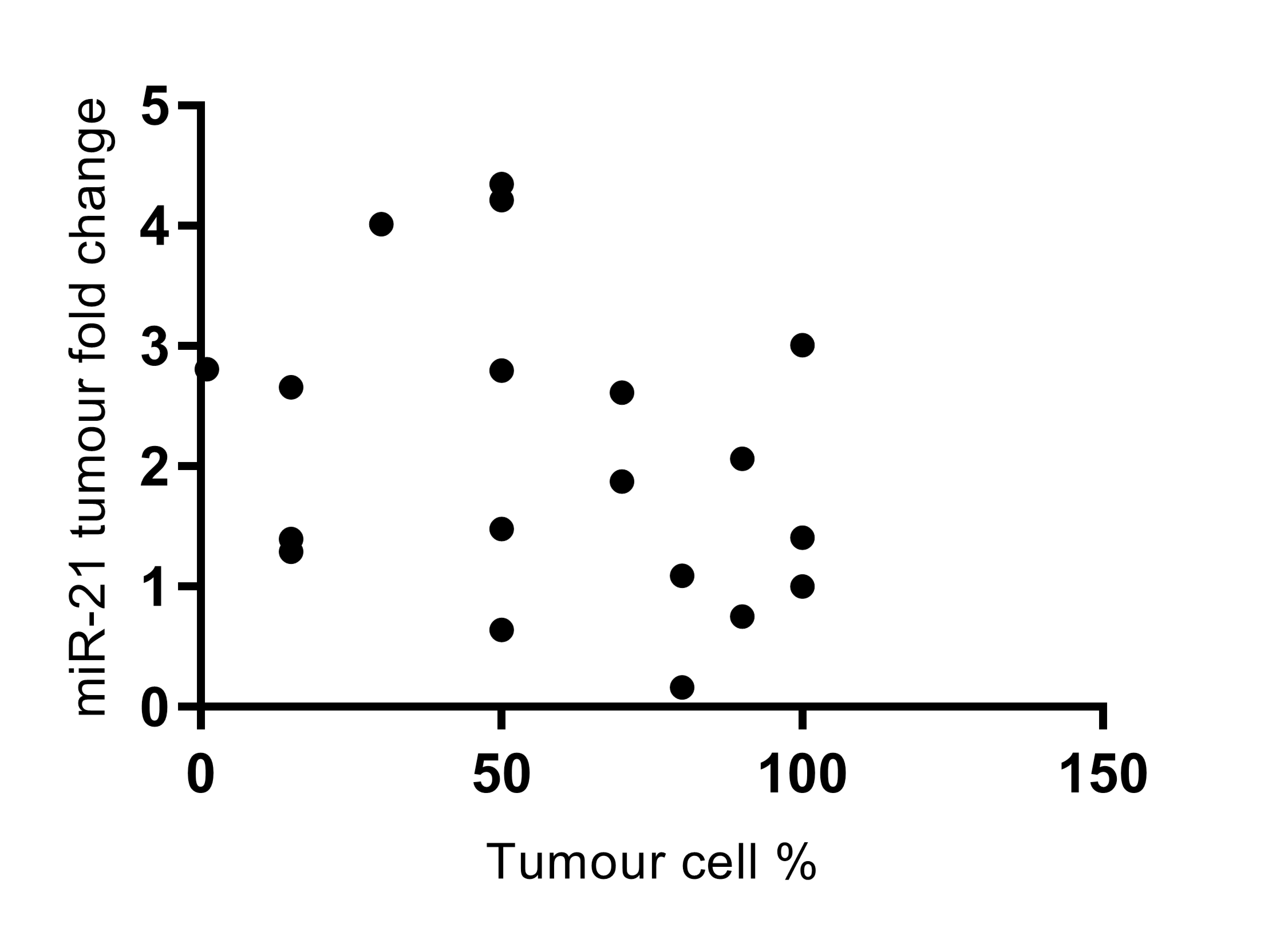

Supplement: S2 Fig — r2 = 0.094, p = 0.20, Pearson correlation. (TIF) [file pone.0249197.s002.tif]

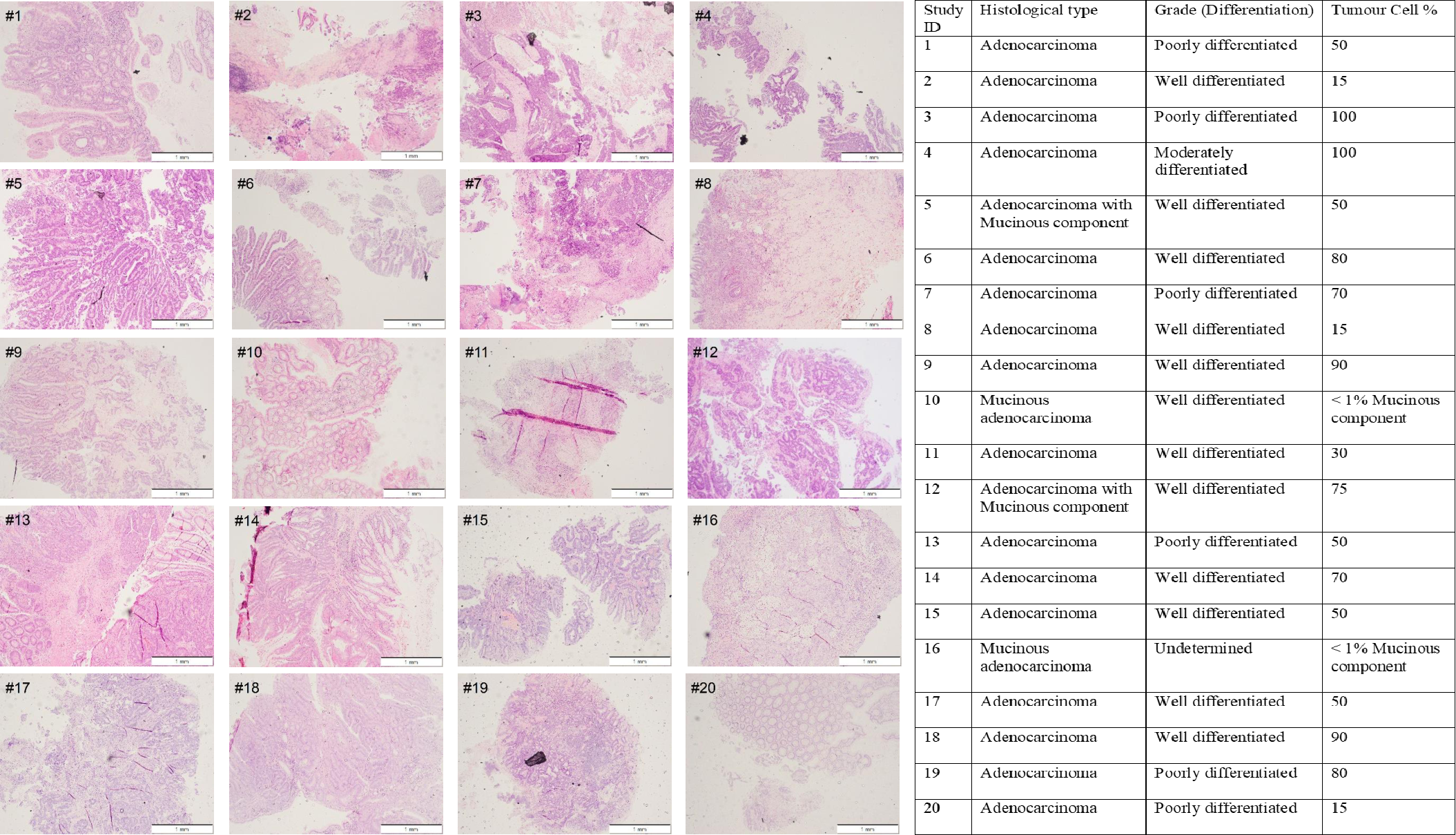

Supplement: S3 Fig — Scale bar = 500 μM. (TIF) [file pone.0249197.s003.tif]
