# Supplementary material for: Brief report: Lymph node morphology in stage II colorectal cancer
Source: PLoS One. 2021 Mar 29;16(3):e0249197. doi: 10.1371/journal.pone.0249197 (PMC8007027; doi:10.1371/journal.pone.0249197)
Supplement: S3 Table — (DOCX) [file pone.0249197.s006.docx]

**S3 Table. Demographics, clinicopathological features and TDLN histomorphology assessed by individual lymph nodes.**

|  | F density | | GC density | | Primary Follicle density | | F size | | GC size | | Marginal zone | |
| --- | --- | --- | --- | --- | --- | --- | --- | --- | --- | --- | --- | --- |
|  | Median | p value | Median | p value | Median | p value | Median | p value | Median | p value | Median | p value |
| Female | 12 (8-16) | 0.54 | 7.5 (5-12) | 0.63 | 3 (1-5) | 0.45 | 0.111 (0.073-0.170) | 0.12 | 0.036 (0.017-0.072) | 0.23 | 0.073 (0.052-0.110) | 0.26 |
| Male | 12 (7-16) |  | 8 (4-12) |  | 3 (1-5) |  | 0.119 (0.089-0.183) |  | 0.040 (0.025-0.076) |  | 0.083 (0.060-0.098) |  |
| Age >66 | 12 (8-16) | 0.56 | 8 (5-12) | 0.67 | 2 (1-5) | 0.40 | 0.103 (0.072-0.181) | 0.21 | 0.034 (0.019-0.076) | 0.83 | 0.072 (0.050-0.100) | **0.04** |
| ≤66 | 12 (8-17) |  | 8 (4-12) |  | 3 (1-5) |  | 0.120 (0.083-0.170) |  | 0.039 (0.022-0.068) |  | 0.082 (0.062-0.101) |  |
| T3 stage | 14 (9-17) | **0.0002** | 9 (5-14) | **0.0005** | 3 (1-5) | 0.66 | 0.102 (0.072-0.151) | **0.0007** | 0.034 (0.018-0.058) | **0.02** | 0.069 (0.053-0.088) | **<0.0001** |
| T4a/b stage | 10 (7-13) |  | 7 (4-10) |  | 3 (1-5) |  | 0.141 (0.085-0.201) |  | 0.044 (0.024-0.093) |  | 0.090 (0.066-0.130) |  |
| Adenocarcinoma | 13 (8-16) | 0.91 | 9 (5-13) | **0.0017** | 2 (1-4) | **<0.0001** | 0.120 (0.084-0.505) | **0.0030** | 0.040 (0.025-0.087) | **<0.0001** | 0.078 (0.057-0.105) | 0.12 |
| Mucinous | 12 (8-16) |  | 6 (4-11) |  | 4 (2-9) |  | 0.095 (0.070-0.148) |  | 0.026 (0.013-0.170) |  | 0.070 (0.051-0.154) |  |
| Well differentiated | 12 (8-17) | **0.04** | 8 (5-12) | 0.77 | 3 (1-5) | **0.02** | 0.109 (0.071-0.169) | **0.0044** | 0.036 (0.021-0.073) | 0.15 | 0.071 (0.053-0.093) | **0.0006** |
| Moderate- poorly differentiated | 10 (7-15) |  | 8 (6-11) |  | 2 (1-4) |  | 0.158 (0.090-0.214) |  | 0.046 (0.026-0.090) |  | 0.084 (0.067-0.124) |  |
| CEA <3.5 µg/L | 11 (7-16) | **0.0027** | 7 (4-11) | **<0.0001** | 3 (2-6) | **0.0015** | 0.118 (0.080-0.168) | 0.80 | 0.035 (0.018-0.066) | 0.20 | 0.078 (0.059-0.110) | 0.023 |
| CEA ≥3.5 µg/L | 14 (10-17) |  | 11 (7-14) |  | 2 (1-4) |  | 0.103 (0.074-0.183) |  | 0.039 (-0.024-0.091) |  | 0.070 (0.052-0.091) |  |
| Lymphovascular or peri-neural invasion | 16 (12-19) | **<0.0001** | 11 (6-14) | **0.038** | 4 (2-7) | **0.0006** | 0.091 (0.065-0.148) | **0.028** | 0.032 (0.014-0.052) | **0.020** | 0.066 (0.052-0.097) | 0.068 |
| No invasion | 11 (8-15) |  | 7 (4-12) |  | 2 (1-5) |  | 0.117 (0.081-0.184) |  | 0.038 (0.023-0.080) |  | 0.077 (0.059-0.105) |  |
| dMMR | 14 (8-18) | **0.0040** | 9 (5-14) | 0.057 | 3 (1-6) | 0.78 | 0.118 (0.076-0.174) | 0.94 | 0.035 (0.021-0.066) | 0.70 | 0.076 (0.054-0.110) | 0.63 |
| No dMMR | 11 (8-15) |  | 7 (4-11) |  | 3 (1-5) |  | 0.112 (0.081-0.170) |  | 0.037 (0.020-0.076) |  | 0.075 (0.057-0.092) |  |
| Multiple polyps | 13 (10-17) | 0.095 | 8 (5-11) | 0.99 | 3 (2-6) | **0.0049** | 0.120 (0.077-0.168) | 0.95 | 0.035 (0.015-0.072) | 0.29 | 0.077 (0.056-0.108) | 0.46 |
| No polyps | 12 (8-16) |  | 8 (4-13) |  | 2 (1-5) |  | 0.112 (0.080-0.174) |  | 0.036 (0.025-0.071) |  | 0.074 (0.056-0.095) |  |
| Tumour miR-21 ≥2 fold change | 12 (8-16) | 0.58 | 7 (4-12) | 0.061 | 3 (1-5) | 0.19 | 0.096 (0.072-0.130) | **<0.0001** | 0.028 (0.016-0.049) | **<0.0001** | 0.068 (0.052-0.086) | **<0.0001** |
| Tumour miR-21 <2 fold change | 12 (8-17) |  | 9 (5-13) |  | 3 (1-5) |  | 0.170 (0.110-0.230) |  | 0.066 (0.034-0.106) |  | 0.098 (0.073-0.128) |  |

Values expressed as Median ± interquartile range. Mann-Whitney U tests were used for non-normally distributed data.
